# Supplementary material for: N6-methyladenosine RNA modification suppresses antiviral innate sensing pathways via reshaping double-stranded RNA
Source: Nat Commun. 2021 Mar 11;12:1582. doi: 10.1038/s41467-021-21904-y (PMC7952553; doi:10.1038/s41467-021-21904-y)
Supplement: Supplementary file 1 — Supplementary Information [file 41467_2021_21904_MOESM1_ESM.pdf]

Supplementary Information

Supplementary Fig.1-8

N^6-methyladenosine RNA modification suppresses antiviral innate sensing pathways via reshaping double-stranded RNA

Weinan Qiu, Qingyang Zhang, Rui Zhang, Yangxu Lu, Xin Wang, Huabin Tian, Ying Yang, Zijuan Gu, Yanan Gao, Xin Yang, Guanshen Cui, Baofa Sun, Yanan Peng, Hongyu Deng, Hua Peng, Angang Yang, Yun-Gui Yang, and Pengyuan Yang

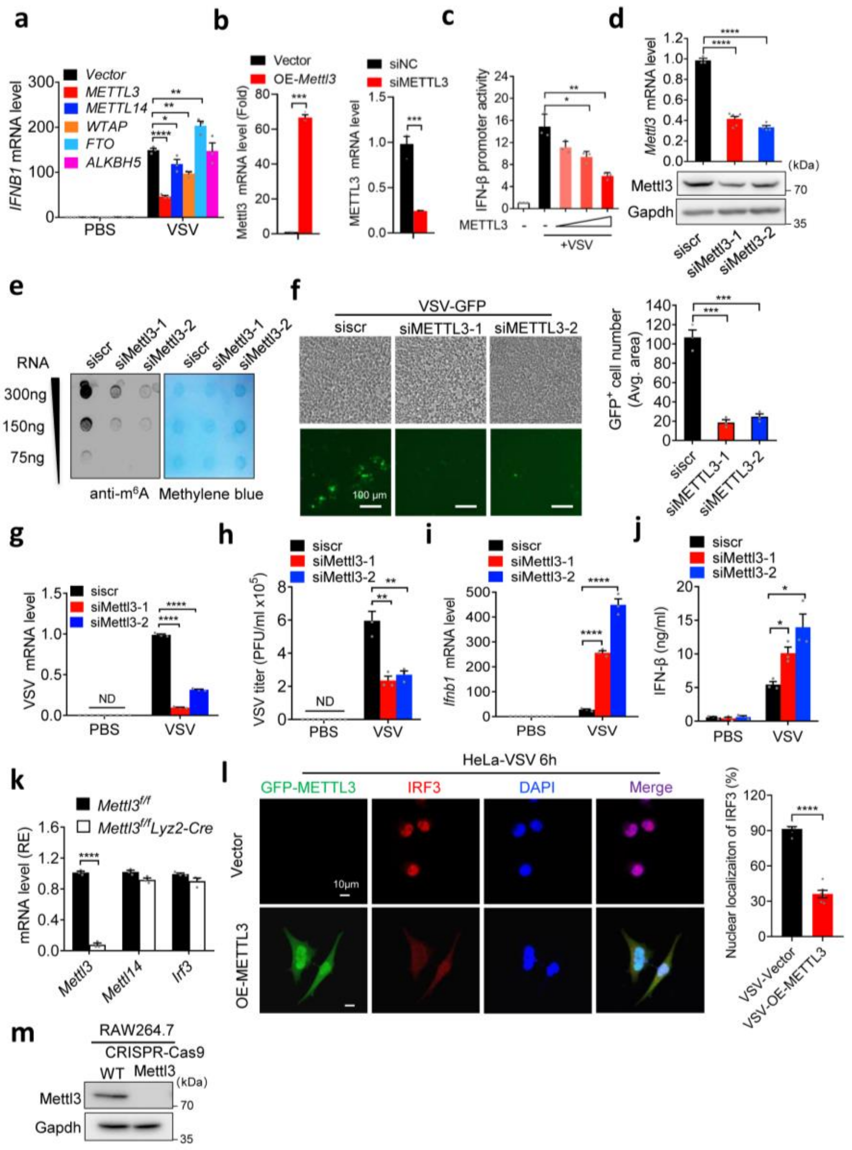

Supplementary Figure 1. METTL3 is a negative regulator in anti-viral innate immunity. **a.** qRT-PCR analysis of *IFNB1* expression following a 12 h treatment with PBS or VSV infection before transfection of different vectors respectively in HeLa cells. **b.** METTL3 overexpression (Left) or knockdown (Right) verified by qPCR. **c.** IFN-β promoter activity in HEK293T cells transfected with gradient of METTL3 vector upon VSV infection. **d.** qPCR (up) and western blot (down) showed transient knockdown of Mettl3 in RAW264.7 cells. **e.** Dot blot analysis of RNA m^{6}A level after transient knockdown of Mettl3 in RAW264.7 cells. ND: not detected. **f.** qPCR analysis of VSV mRNA level after VSV infection for 12 h. **g.** Knockdown of METTL3 in HEK293T cells, then infected with VSV for 18 h. The immunofluorescence indicated VSV-infected cells (left). The bar graph showed statistics (right). **h.** Silenced Mettl3 in RAW264.7 cells and then infected with VSV for 12 h. Collected supernatants performed plaque assay, which indicated the VSV titer. **i. j.** qPCR (i) and ELISA (j) analysis of *Ifnb1*

expression in RAW264.7 cells silenced Mettl3 in mock (PBS) or VSV infection for 12 h. Data are representative of 3 independent experiments. **k.** qPCR analysis of specific depletion of Mettl3 in monocyte of mice. Collected the peritoneal macrophage to extract total RNA for qRT-PCR analysis. **l.** Immunofluorescence analysis of IRF3 translocation and activation in Hela cells. Data are representative of 3 independent experiments. **m.** Western blot analysis showed depletion of Mettl3 in RAW264.7 cells by sgRNA targeting Mettl3. 2 times each experiment was repeated independently with similar results. All the qPCR data in this Figure are representative of 2 independent experiments. *p<0.05, **p<0.01, ***p<0.001, ****p<0.0001 as determined by two-tailed unpaired Student's t test (**a-d, f-l**). Error bars represent mean ± SEM.

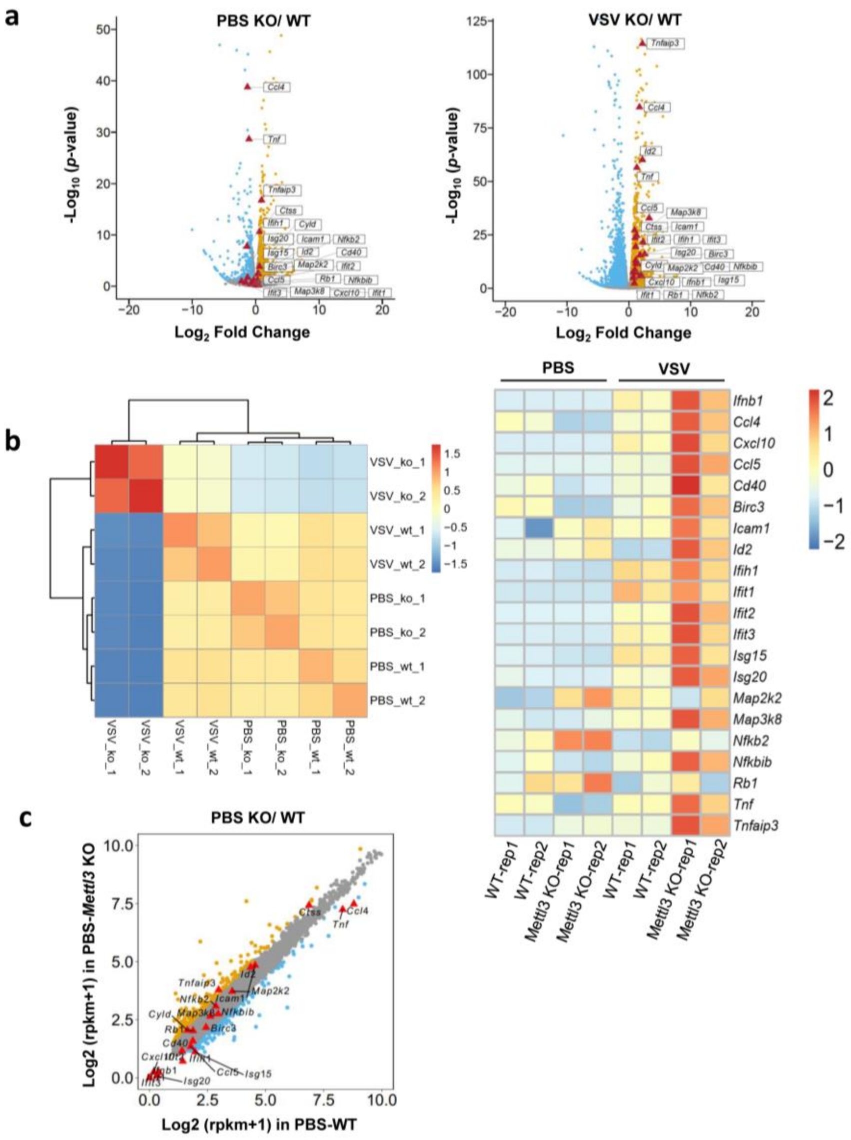

Supplementary Figure 2. Loss of METTL3 upregulates mRNA level of innate immunity genes upon VSV infection.

a. Volcano plots of genes with differential expression in Mettl3 knockout and WT RAW264.7 cells after VSV infection for 12 h. (yellow: up-regulated; blue: down-regulated; grey: no significant change; red triangle: ISGs). **b.** Heatmap depicting the correlation of replicated samples along different conditions (left panel). Heatmap showing the expression of innate immunity related genes in knockout RAW264.7 cells versus wild type cells (right panel). **c.** Scatter plot showing the alteration of genes expression by comparing Mettl3 knockout and WT RAW264.7 cells under PBS treatment as mock controls (yellow, up-regulated; blue, down-regulated; grey, no significant change; red triangle, ISGs).

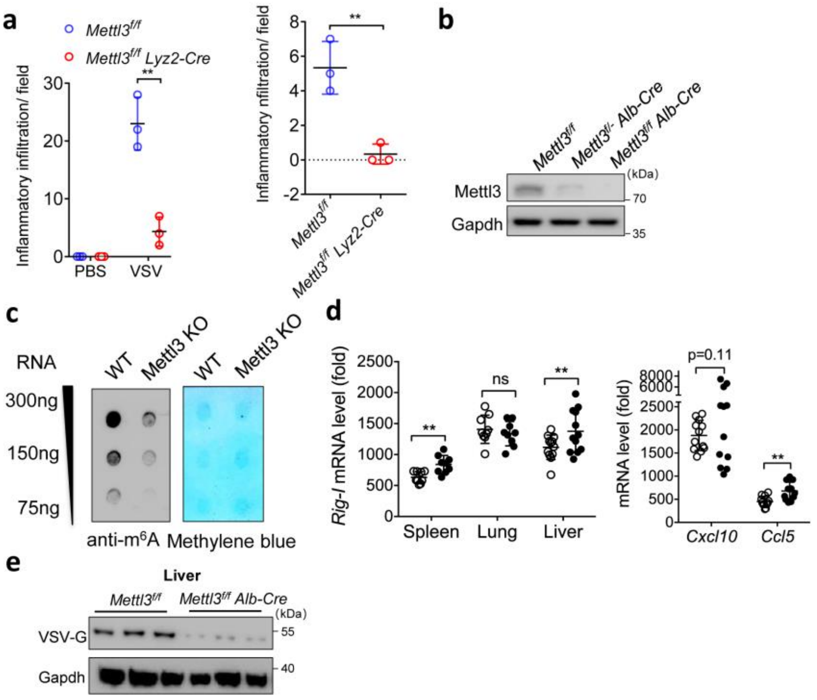

Supplementary Figure 3. METTL3 inhibits anti-viral ability *in vivo*.

a. The statistics of Fig. 2b (Left) and Fig. 2c (Right). n=3 biologically independent animals. **b.** Western blot analysis of depletion of Mettl3 in primary hepatocyte. **c.** RNA dot blot analysis of m^{6}A level in hepatocytes from WT or Mettl3 KO mice. **d.** qPCR analysis of ISGs mRNA in *Mettl3$^{f/f}$ Alb*-Cre and *Mettl3$^{f/f}$* mice (n = 5 per group) given intraperitoneal injection of PBS or infected for 24 h by intraperitoneal injection of VSV (2.5 × 10^8 PFU per mouse); results are presented relative to those of *actin.* **e.** Western blot analysis of VSV-G in the livers of infected mice. Data are representative of 2-3 independent experiments. *p<0.05, **p<0.01 as determined by two-tailed unpaired Student's t test (**d, e**). Error bars represent mean ± SEM.

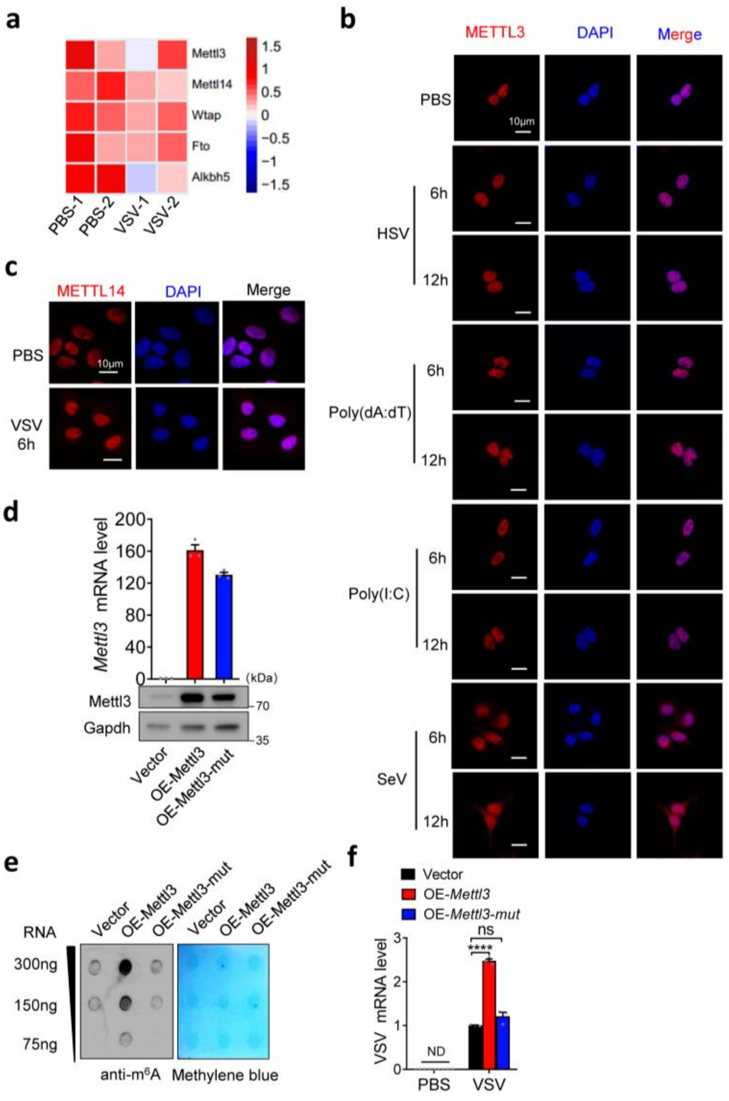

Supplementary Figure 4. RNA virus induces METTL3 cytosolic translocation.

a. Heatmap showed RNA m^{6}A editing genes expression in RAW264.7 cells upon PBS or VSV treatment. **b.** Immunofluorescent results indicate the endogenous METTL3 localization upon different treatments at indicated timepoints in HeLa cells. 2 times each experiment was repeated independently with similar results. **c.** Immunofluorescent result indicates the endogenous METTL14 localization upon VSV treatment for 6 h. 2 times each experiment was repeated independently with similar results. **d.** qPCR (up) and western blot (down) showed overexpression of Mettl3 or Mettl3-mut in RAW264.7 cells. Data are representative of 2 independent experiments. **e.** Dot blot analysis of RNA m^{6}A level after overexpression of Mettl3 or Mettl3-mut in RAW264.7 cells. **f.** qPCR analysis of VSV mRNA level after VSV infection for 12 h. Data are representative of 2 independent experiments. ****$p<0.0001$ as determined by two-tailed unpaired Student's *t* test (**f**). Error bars represent mean ± SEM.

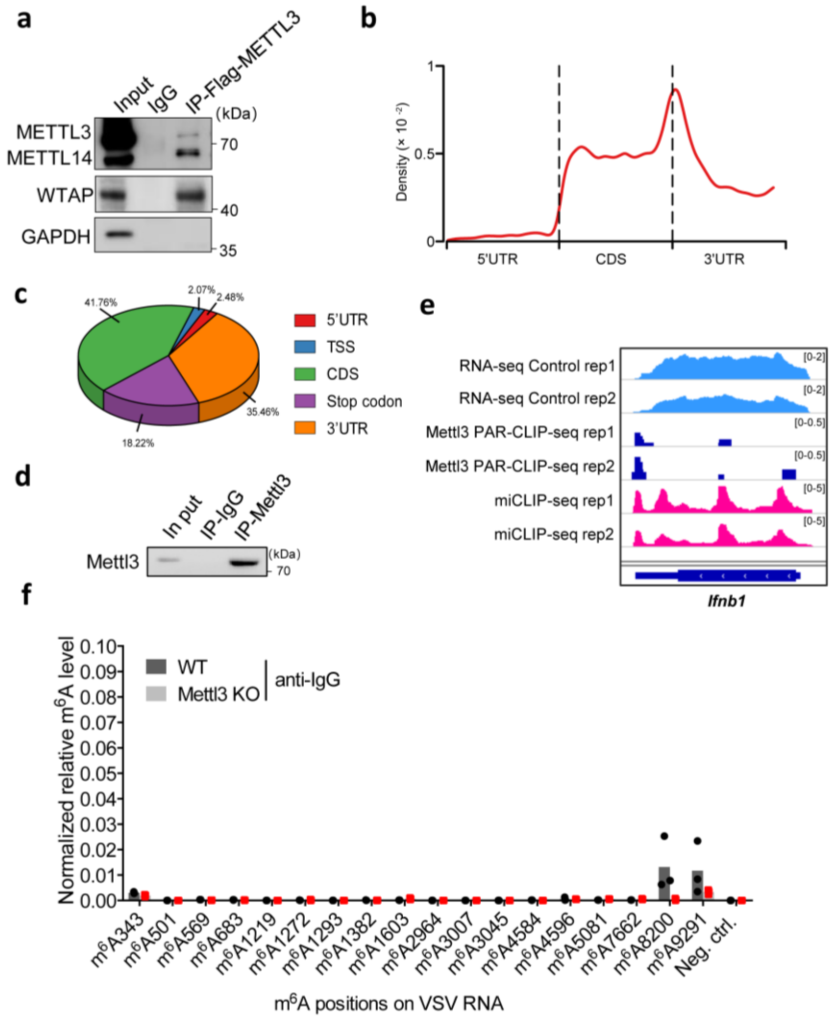

Supplementary Figure 5. *IFN-β1* mRNA contains m^{6}A modification.

a. Western blot analysis indicated the efficiency of immunoprecipitation. Transient transfection of Flag-METTL3 in HEK293T cells before performed anti-Flag or anti-IgG immunoprecipitation. **b.** Distribution of m^{6}A peaks along transcripts in RAW264.7 cell samples. **c.** Pie chart showed the percentage of m^{6}A peaks located in CDS, UTR, TSS (in the first 200 nucleotides of a transcript) and stop codon (400-nucleotide window centred on the stop codon). **d.** IP-western blot assay for Mettl3 protein by using RAW264.7 cell lysate to show the specificity of Mettl3 antibody. IgG as negative control. **e.** Sites of METTL3-binding and m^{6}A modification on *Ifnb1* mRNA identified by miCLIP-seq and PAR-CLIP respectively in RAW264.7 cells. RNA-seq result served as input control. **f.** MeRIP-qPCR analysis of specific m^{6}A sites on VSV RNA in WT or Mettl3 KO RAW264.7 cells. The bar graph showed anti-IgG control. Data are representative of 2-3 independent experiments.

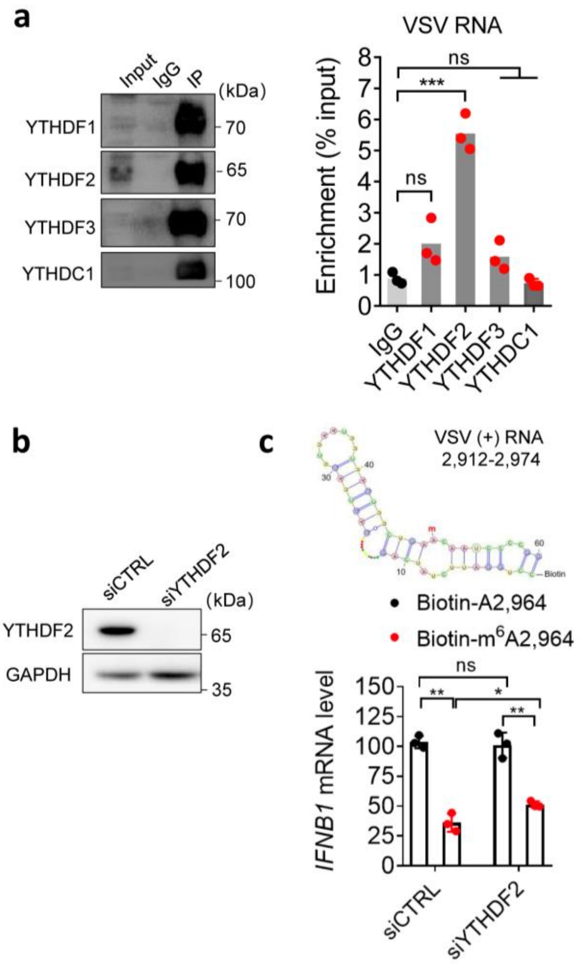

Supplementary Figure 6. Analysis of m^{6}A reader proteins in VSV-induced innate immunity.

a. RIP-qPCR was performed by HeLa cell lysates. Western blot indicated the m^{6}A "readers" antibodies are suitable to carry out immunoprecipitation. 2 times each experiment was repeated independently with similar results. **b.** Western blot indicated the efficiency of knockdown of YTHDF2 by siRNA in HeLa cells. 2 times each experiment was repeated independently with similar results. **c.** The HeLa cells were transfected with VSV (+) RNA oligoes (biotin-A2,964 and biotin-m^{6}A2,964) respectively for 12h, and then extracted RNA to perform qPCR assay. *p<0.05, **p<0.01, ***p<0.001 as determined by two-tailed unpaired Student's t test (**a, c**). Error bars represent mean ± SEM.

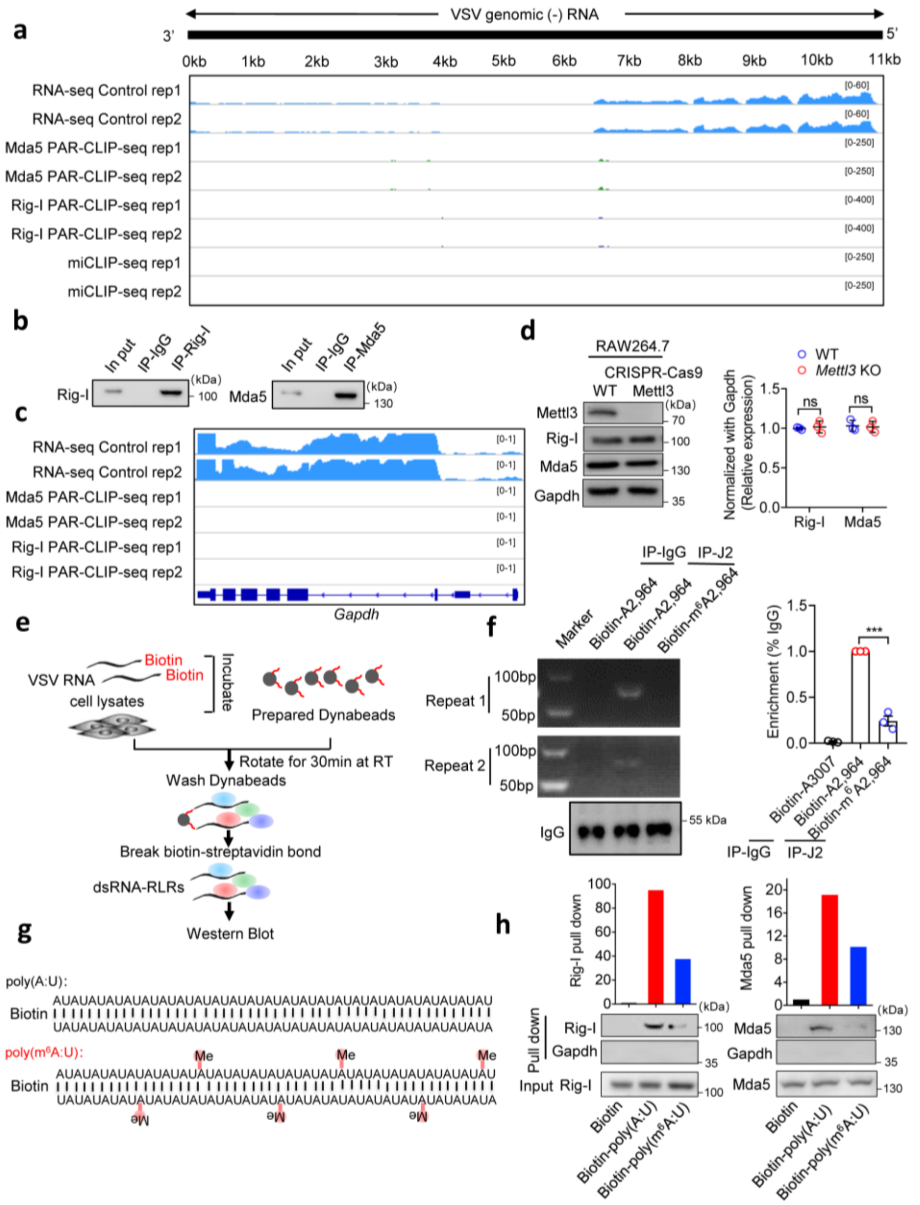

Supplementary Figure 7. m^{6}A modification impairs RLRs sensing for VSV dsRNA.

a. Integrative genomics viewer (IGV) plots the m^{6}A sites and RIG-I and MDA5-binding regions on VSV (-) RNA. RNA-seq data were used as input control. **b.** IP-western blot assay for Rig-I and Mda5 proteins by using RAW264.7 cell lysate to show the specificity of Mettl3 antibody. IgG as negative control. Data are representative of 2 independent experiments. **c.** Sites of Mda5 and Rig-I binding on *Gapdh* mRNA (as negative control) in RAW264.7 cells. RNA-seq result served as input control. **d.** Western blot indicated loss of Mettl3 did not change the expression of Mda5 and Rig-I protein level. The right panel indicates statistic result. Data are representative of 3 independent experiments. **e.** A schematic representation of the experimental procedure used in Fig. 6c and Fig. S6c. **f.** Performed anti-dsRNA (J2)-IP and then run nuclear acid gel to detect the binding affinity between J2 antibody and synthesized biotin-labeled viral

RNAs +/- m^{6}A. IgG bands indicated loading control. Data are representative of 3 independent experiments. **g**. Artificially synthesized biotin-labeled poly(A:U) and poly(m^{6}A:U). **h.** Biotin-labeled RNA pull-down and western blot analysis of Rig-I and Mda5 bindings to RNA oligo with or without m^{6}A modification. Histograms show relative density of western blot bands (up). *p<0.05, **p<0.01, ***p<0.001 as determined by two-tailed unpaired Student's *t* test (**d, f**). Error bars represent mean ± SEM.

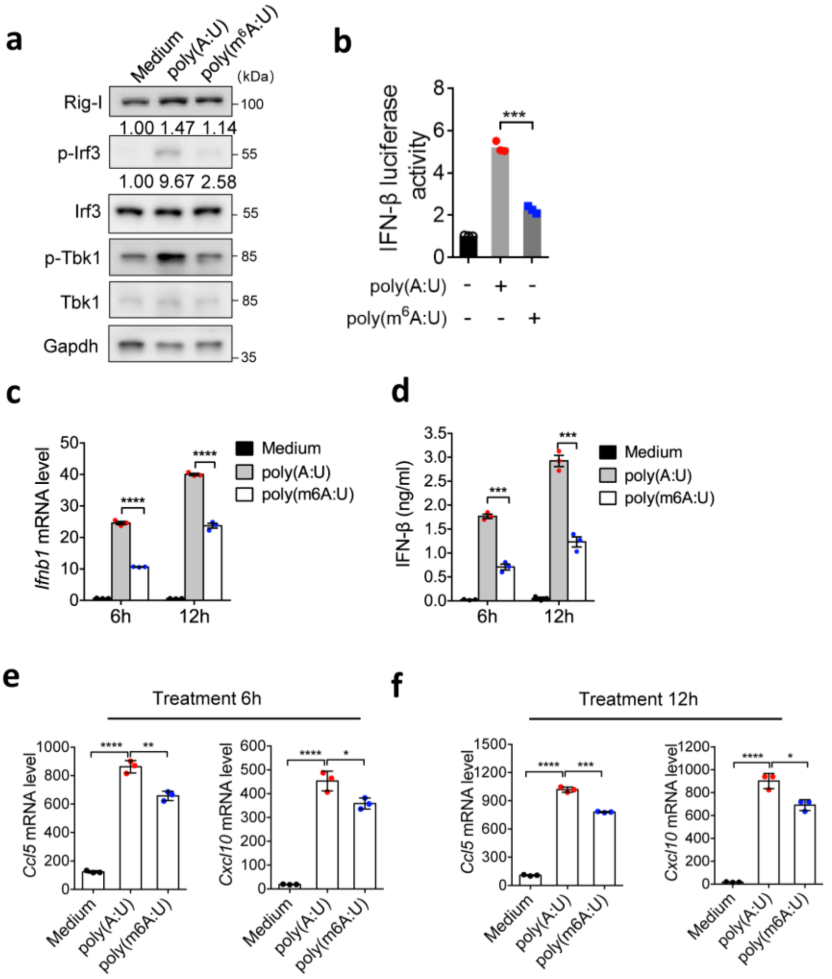

Supplementary Figure 8. m^{6}A modification on dsRNA oligo impedes IRF3-IFN-β-ISGs pathway.

a. Western blot showed that m^{6}A modification limited poly(A:U)-induced Tbk1-Irf3 activation. **b.** IFN-β promoter activity in HEK293T cells transfected with poly(A:U) or poly(m^{6}A:U). **c. d.** qPCR (**c**) and ELISA (**d**) showed that m^{6}A modification limited poly(A:U)-induced *Ifnb1* expression and secretion. **e. f.** qPCR analysis showed that m^{6}A modification limited poly(A:U)-induced ISGs-*Ccl5, Cxcl10* expression. **p*<0.05, ***p*<0.01, ****p*<0.001, *****p*<0.0001 as determined by two-tailed unpaired Student's *t* test (**b-f**). Error bars represent mean ± SEM.
