# Supplementary material for: N6-methyladenosine RNA modification suppresses antiviral innate sensing pathways via reshaping double-stranded RNA
Source: Nat Commun. 2021 Mar 11;12:1582. doi: 10.1038/s41467-021-21904-y (PMC7952553; doi:10.1038/s41467-021-21904-y)
Supplement: Supplementary file 3 — Reporting Summary [file 41467_2021_21904_MOESM3_ESM.pdf]

nature research

Corresponding author(s): Pengyuan Yang; Yun-Gui Yang

Last updated by author(s): 2021/01/31

# Reporting Summary

Nature Research wishes to improve the reproducibility of the work that we publish. This form provides structure for consistency and transparency in reporting. For further information on Nature Research policies, see our Editorial Policies and the Editorial Policy Checklist.

## Statistics

For all statistical analyses, confirm that the following items are present in the figure legend, table legend, main text, or Methods section.

| n/a | Confirmed | |
|---|---|---|
| ☐ | ☒ | The exact sample size (*n*) for each experimental group/condition, given as a discrete number and unit of measurement |
| ☐ | ☒ | A statement on whether measurements were taken from distinct samples or whether the same sample was measured repeatedly |
| ☐ | ☒ | The statistical test(s) used AND whether they are one- or two-sided<br>*Only common tests should be described solely by name; describe more complex techniques in the Methods section.* |
| ☒ | ☐ | A description of all covariates tested |
| ☐ | ☒ | A description of any assumptions or corrections, such as tests of normality and adjustment for multiple comparisons |
| ☐ | ☒ | A full description of the statistical parameters including central tendency (e.g. means) or other basic estimates (e.g. regression coefficient) AND variation (e.g. standard deviation) or associated estimates of uncertainty (e.g. confidence intervals) |
| ☐ | ☒ | For null hypothesis testing, the test statistic (e.g. *F*, *t*, *r*) with confidence intervals, effect sizes, degrees of freedom and *P* value noted<br>*Give P values as exact values whenever suitable.* |
| ☒ | ☐ | For Bayesian analysis, information on the choice of priors and Markov chain Monte Carlo settings |
| ☒ | ☐ | For hierarchical and complex designs, identification of the appropriate level for tests and full reporting of outcomes |
| ☒ | ☐ | Estimates of effect sizes (e.g. Cohen's *d*, Pearson's *r*), indicating how they were calculated |

*Our web collection on statistics for biologists contains articles on many of the points above.*

## Software and code

Policy information about availability of computer code

| | |
|---|---|
| Data collection | For imaging on Zeiss LSM700 microscope: Zeiss ZEN2012; Image J (V 1.52v) (https://imagej.en.softonic.com) was used to analyze gel intensity and figures; Graph pad version 8.0.1 (https://www.graphpad.com) was used to generate bar graphs and statistical analysis; igvtools was used to show the sequencing results. |
| Data analysis | RNA-seq:<br>1) FastQC (v0.11.5) to check the data quality of raw sequencing data.<br>2) hisat2 (v2.0.5) for reads alignment.<br>3) FeatureCounts (v1.6.0) to calculate the read counts of per gene.<br>4) DESeq2 (v3.18.1) to do the differential gene expression analysis.<br>5) ToppGene Suite (https://toppgene.cchmc.org/) for GO enrichment and Reactome pathway enrichment.<br>6) GSEA software (http://www.broad.mit.edu/GSEA) for Gene set enrichment analysis.<br>miCLIP-seq:<br>1) FASTX-Toolkit (http://hannonlab.cshl.edu/fastx_toolkit) to remove the adaptor.<br>2) CTK Tool Kit (v1.0.3) fastq2collapse.pl to remove PCR-amplified reads.<br>3) Cutadapt (v1.16) to trim the polyA-tail.<br>4) CTK Tool Kit (v1.0.3) stripBarcode.pl to remove the random barcode.<br>5) Trimmomatic (v0.33) to remove the short reads (< 18nt).<br>6) BWA (v0.7.17-r1188) for data aligenment.<br>7) CTK Tool Kit (v1.0.3) to detect the m6A sites.<br>8) Weblogo3 for motif generation.<br>9) Integrative Genomics Viewer (IGV) to visualize the m6A distribution.<br>10) genomeCoverageBed from bedtools (v2.26.0) to transform alignments into bedGraph file. |

11) bedGraphToBigWig (v4) to convert into bigwig format file.
PAR-CLIP-seq:
1) Cutadapt (v1.16) to remove adaptor.
2) bowtie(v1.0.1) for data alignment.
3) PARalyzer (v1.5) to detect the binding peak.
Image J were used for confocal images analysis; Graphpad were used for bar graphs output and statistic analysis; The software and algorithms for data analyses used in this study are all well-established from previous work. All software and custom arguments are included in Methods section. There is no unreported algorithm used in this paper. The source code for data processing are available from the corresponding author on reasonable request.

For manuscripts utilizing custom algorithms or software that are central to the research but not yet described in published literature, software must be made available to editors and reviewers. We strongly encourage code deposition in a community repository (e.g. GitHub). See the Nature Research guidelines for submitting code & software for further information.

## Data

Policy information about availability of data

All manuscripts must include a data availability statement. This statement should provide the following information, where applicable:

- Accession codes, unique identifiers, or web links for publicly available datasets
- A list of figures that have associated raw data
- A description of any restrictions on data availability

The raw data can be accessed from Genome Sequence Archive with accession number: CRA002259. Besides, the original data is also available at Sequence Read Archive with number PRJNA634708. The raw numbers for charts and graphs are available in the Source Data file whenever possible. All other data supporting the findings of this study are available from the corresponding author on reasonable request.

# Field-specific reporting

Please select the one below that is the best fit for your research. If you are not sure, read the appropriate sections before making your selection.

[x] Life sciences [ ] Behavioural & social sciences [ ] Ecological, evolutionary & environmental sciences

For a reference copy of the document with all sections, see nature.com/documents/nr-reporting-summary-flat.pdf

# Life sciences study design

All studies must disclose on these points even when the disclosure is negative.

| | |
|---|---|
| Sample size | For animal experiments, n=5 and performed 2 independent experimental repeats. Sample sizes were determined by power analysis, with mean and deviation estimated from preliminary studies. |
| Data exclusions | No data were excluded from the analysis. |
| Replication | All findings reported were reproducible and data shown are pooled from >=2 independent experiments, with comparable results in each experiment. |
| Randomization | For cell experiments, all cells in each experiment were from the same pool of parental cells. For animal experiments, all mice were age- and sex-matched (littermate female mice) and then randomized into different experimental groups. All animals were maintained in the same environment and handled by the same procedure. |
| Blinding | For cell-based experiments, Western blotting, immunostaining and qPCR, cell types were known when prepare the samples or start to treat cells at the beginning of experiments. Data measurement were blinded to different person who processed assay at the time. miCLIP-seq, RNA-seq and PAR-CLIP-seq analysis were blinded before analysis. |

# Reporting for specific materials, systems and methods

We require information from authors about some types of materials, experimental systems and methods used in many studies. Here, indicate whether each material, system or method listed is relevant to your study. If you are not sure if a list item applies to your research, read the appropriate section before selecting a response.

## Materials & experimental systems

| n/a | Involved in the study |
| --- | --- |
| | ☒ Antibodies |
| | ☒ Eukaryotic cell lines |
| ☒ | ☐ Palaeontology and archaeology |
| | ☒ Animals and other organisms |
| ☒ | ☐ Human research participants |
| ☒ | ☐ Clinical data |
| ☒ | ☐ Dual use research of concern |

## Methods

| n/a | Involved in the study |
| --- | --- |
| ☒ | ☐ ChIP-seq |
| ☒ | ☐ Flow cytometry |
| ☒ | ☐ MRI-based neuroimaging |

# Antibodies

Antibodies used

GAPDH (Cell Signaling Technology, CAT#5174S); TBK1 (NOVUS, CAT#NB100-56705); IRF3 (NOVUS, CAT#NBP2-67741); RIG-I (ABGENT, CAT#AP1900A); VSV-G (ABGENT, CAT#AP1016a); TLR3 (Proteintech, CAT#17766-1-AP); TBK1 (Abcam, CAT#ab40676); MDA5 (Abcam, CAT#ab79055); METTL3 (Bethyl, CAT#A301-567A); TRAF3 (Cell Signaling Technology, CAT#4729T); TRAF6 (Cell Signaling Technology, CAT#8028T); TBK1 (Cell Signaling Technology, CAT#3504T); p-TBK1 (Cell Signaling Technology, CAT#5483T); Rig-I (Cell Signaling Technology, CAT#3743T); LaminA/C (Cell Signaling Technology, CAT#4777T); IRF3 (Cell Signaling Technology, CAT#4302S); p-IRF3 (Cell Signaling Technology, CAT#29047S); p-stat3 (Cell Signaling Technology, CAT#9145S); p65  (Cell Signaling Technology, CAT#6956S); p-p65 (Cell Signaling Technology, CAT#3303S); p38 (Cell Signaling Technology, CAT#14451S); p-p38 (Cell Signaling Technology, CAT#4511S); p-stat1 (Abcam, CAT#ab109461); J2(dsRNA) (SCICONS, CAT#J2-1809); YTHDF1 (Proteintech, CAT#17479-1-AP); YTHDF2 (Proteintech, CAT#24744-1-AP); YTHDF3 (Proteintech, CAT#25537-1-AP); YTHDC1 (Proteintech, CAT#14392-1-AP); MDA5 (Cell Signaling Technology, CAT#5321); RIG-I (Cell Signaling Technology, CAT#4200); WTAP (Proteintech, CAT#10200-1-AP); METTL14 (SIGMA, CAT#HPA038002); MAVS (Proteintech, CAT#14341-1-AP); m6A (Synaptic Systems, CAT# 202003).

Validation

All antibodies used in our study have been validated and detailed information could be found on the website from manufactures as listed below. Some of them have also been validated by our experiments as shown in this manuscript using either overexpress, knockout or knockdown strategies.

GAPDH https://www.cellsignal.com/products/primary-antibodies/gapdh-d16h11-xp-rabbit-mab/5174?site-search-type=Products&N=4294956287&Ntt=5174s&fromPage=plp&_requestid=2283733;
TBK1 https://www.novusbio.com/products/tbk1-antibody-108a429_nb100-56705;
IRF3 https://www.novusbio.com/products/irf3-antibody-sd2062_nbp2-67741;
RIG-I http://www.abcepta.com/products/AP1900a-RIG-I-Antibody-C-term;
VSV-G http://www.abcepta.com/products/AP1016a-VSV-g-Tag-Antibody;
TLR3 http://www.ptgcn.com/products/TLR3-Antibody-17766-1-AP.htm;
TBK1 https://www.abcam.cn/naktbk1-antibody-ep611y-ab40676.html;
MDA5 https://www.abcam.cn/mda5-antibody-ab79055.html;
METTL3 https://www.bethyl.com/product/A301-567A?referrer=search;
TRAF3 https://www.cellsignal.com/products/primary-antibodies/traf3-antibody/4729?site-search-type=Products&N=4294956287&Ntt=4729t&fromPage=plp&_requestid=2412838;
TRAF6 https://www.cellsignal.com/products/primary-antibodies/traf6-d21g3-rabbit-mab/8028;
TBK1 https://www.cellsignal.com/products/primary-antibodies/tbk1-nak-d1b4-rabbit-mab/3504;
p-TBK1 https://www.cellsignal.com/products/primary-antibodies/phospho-tbk1-nak-ser172-d52c2-xp-rabbit-mab/5483;
RIG-I https://www.cellsignal.com/products/primary-antibodies/rig-i-d14g6-rabbit-mab/3743;
LaminA/C https://www.cellsignal.com/products/primary-antibodies/lamin-a-c-4c11-mouse-mab/4777;
IRF3 https://www.cellsignal.com/products/primary-antibodies/irf-3-d83b9-rabbit-mab/4302;
p-IRF3 https://www.cellsignal.com/products/primary-antibodies/phospho-irf-3-ser396-d6o1m-rabbit-mab/29047;
p-STAT3 https://www.cellsignal.com/products/primary-antibodies/phospho-stat3-tyr705-d3a7-xp-rabbit-mab/9145;
p65 https://www.cellsignal.com/products/primary-antibodies/nf-kb-p65-l8f6-mouse-mab/6956;
p-p65 https://www.cellsignal.com/products/primary-antibodies/phospho-nf-kb-p65-ser536-93h1-rabbit-mab/3033?site-search-type=Products;
p38 https://www.cellsignal.com/products/antibody-conjugates/p38-mapk-d13e1-xp-rabbit-mab-hrp-conjugate/14451;
p-p38 https://www.cellsignal.com/products/primary-antibodies/phospho-p38-mapk-thr180-tyr182-d3f9-xp-rabbit-mab/4511;
p-STAT1 https://www.abcam.cn/stat1-phospho-s727-antibody-epr3146-ab109461.html;
J2 (dsRNA) https://scicons.eu/en/antibodies/j2/;
YTHDF1 https://www.ptgcn.com/Products/YTHDF1-Antibody-17479-1-AP.htm;
YTHDF2 https://www.ptgcn.com/Products/YTHDF2-Antibody-24744-1-AP.htm;
YTHDF3 https://www.ptgcn.com/Products/YTHDF3-Antibody-25537-1-AP.htm;
YTHDC1 https://www.ptgcn.com/Products/YTHDC1-Antibody-14392-1-AP.htm;
MDA5 https://www.cellsignal.com/products/primary-antibodies/mda-5-d74e4-rabbit-mab/5321;
RIG-I https://www.cellsignal.com/products/primary-antibodies/rig-i-d33h10-rabbit-mab/4200;
WTAP https://www.ptgcn.com/Products/WTAP-Antibody-10200-1-AP.htm;
METTL14 https://www.sigmaaldrich.com/catalog/product/sigma/hpa038002?lang=zh&region=CN;
MAVS https://www.ptgcn.com/products/MAVS-Antibody-14341-1-AP.htm;
m6A https://www.sysy.com/products/m6a/facts-202003.php.

## Eukaryotic cell lines

Policy information about cell lines

| | |
|---|---|
| Cell line source(s) | RAW264.7 cell line, Vero cell line, HEK293T cell line, LO2 cell line, HeLa cell line, A549 cell line, AC12 cell line and Huh7 cell line were purchased from ATCC. |
| Authentication | Cell lines were not authenticated. |
| Mycoplasma contamination | All cell lines tested negative for mycoplasma contamination. |
| Commonly misidentified lines (See ICLAC register) | No commonly misidentified lines were used in this study. |

## Animals and other organisms

Policy information about studies involving animals; ARRIVE guidelines recommended for reporting animal research

| | |
|---|---|
| Laboratory animals | Mettl3 floxed mice, gifted from Dr. Rui Zhang (Fourth Military Medical University), were generated by Beijing Biocytogen Co., Ltd. Alb-Cre mouse strain (The Jackson Lab Stock NO.: 016832) was gifted from Dr. Lijian Hui (Shanghai Institute for Biological Sciences, CAS) and Lyz2-Cre mouse strain (The Jackson Lab Stock NO.: 004781) was gifted from Dr. Mingzhao Zhu (Institute of Biophysics, CAS). We generated Mettl3flox/flox Alb-Cre and Mettl3flox/flox Lyz2-Cre mice by crossing Mettl3flox/flox mice with Alb-Cre and Lyz2-Cre mice respectively. These animals were maintained in the Animal Facilities of Institute of Biophysics, Chinese Academy of Sciences. Mice were maintained in a specific pathogen free unit on a 12 h light: 12 h dark cycle. The animal rooms are provided with 100% fresh, HEPA filtered air at 10-15 air changes per hour. Room temperatures are controlled by reheat units within each room, and are maintained within the range of 70°F ± 2° F. The Humidity levels are controlled globally, and it is maintained between 30-70%. All experimental and control mice were co-housed. And euthanasia was performed for all the animals in this study. All investigations involving mice were approved by the Animal Care and Use Committee of Institute of Biophysics, Chinese Academy of Sciences. |
| Wild animals | No wild animals were used. |
| Field-collected samples | No field-collected samples were used in this study. |
| Ethics oversight | All investigations involving mice were approved by the Animal Care and Use Committee of Institute of Biophysics, Chinese Academy of Sciences. |

Note that full information on the approval of the study protocol must also be provided in the manuscript.
