# Supplementary material for: N6-methyladenosine RNA modification suppresses antiviral innate sensing pathways via reshaping double-stranded RNA
Source: Nat Commun. 2021 Mar 11;12:1582. doi: 10.1038/s41467-021-21904-y (PMC7952553; doi:10.1038/s41467-021-21904-y)
Supplement: Supplementary file 4 — Description of Additional Supplementary Files [file 41467_2021_21904_MOESM4_ESM.pdf]

Description of Additional Supplementary Files

File Name: Supplementary Data 1

Description: **Antibodies information**

File Name: Supplementary Data 2

Description: **Cell Lines information**

File Name: Supplementary Data 3

Description: **Oligonucleotides sequences information**

File Name: Supplementary Data 4

Description: **Plasmids information**

File Name: Supplementary Data 5

Description: **ELISA Kit information**
